# Target Profile Prediction and Practical Evaluation of a Biginelli-Type Dihydropyrimidine Compound Library

**DOI:** 10.3390/ph4091236

**Published:** 2011-09-20

**Authors:** Petra Schneider, Katharina Stutz, Ladina Kasper, Sarah Haller, Michael Reutlinger, Felix Reisen, Tim Geppert, Gisbert Schneider

**Affiliations:** Swiss Federal Institute of Technology (ETH), Department of Chemistry and Applied Biosciences, Institute of Pharmaceutical Sciences, Wolfgang-Pauli-Str. 10, CH-8093 Zurich, Switzerland

**Keywords:** combinatorial chemistry, drug design, *in silico* pharmacology, kinase inhibitor, multi-component reaction, self-organizing map

## Abstract

We present a self-organizing map (SOM) approach to predicting macromolecular targets for combinatorial compound libraries. The aim was to study the usefulness of the SOM in combination with a topological pharmacophore representation (CATS) for selecting biologically active compounds from a virtual combinatorial compound collection, taking the multi-component Biginelli dihydropyrimidine reaction as an example. We synthesized a candidate compound from this library, for which the SOM model suggested inhibitory activity against cyclin-dependent kinase 2 (CDK2) and other kinases. The prediction was confirmed in an *in vitro* panel assay comprising 48 human kinases. We conclude that the computational technique may be used for ligand-based *in silico* pharmacology studies, *off*-target prediction, and drug re-purposing, thereby complementing receptor-based approaches.

## 1. Introduction

Combinatorial fragment-based approaches have become state-of-the-art in computer-assisted lead identification and drug design, with many successful case studies reported [1-3]. Recently, the reaction-based enumeration of virtual compounds has been successfully applied to *de novo* design [4,5]. This concept is thought to result in chemically meaningful and synthetically feasible compounds with desired properties. Following this design concept, two pivotal questions must be answered prior to the virtual synthesis of potentially bioactive compounds, namely: (i) which reaction scheme(s); and (ii) which molecular representation(s) are suited for a given molecular design task? Multi-component reactions and pharmacophore feature representations have been broadly applied in both computational and practical drug design studies [6,7]. For example, a large array of four-component Ugi-reaction products was investigated for serine protease inhibition [8], and three-component Ugi-type products served as an early proof-of-concept study using a genetic algorithm for compound optimization [9].

Reaction-driven, fragment-based *de novo* design of bioactive compounds starts from a set of molecular building blocks and one or more suitable reactions for virtual product formation [10]. The actual fragment assembly step is carried out *in silico*, where two strategies may be pursued: (i) stepwise fragment assembly and iterative optimization of the virtual products; or (ii) full virtual library enumeration and exhaustive screening. The first approach is preferable when very large combinatorial compound libraries prohibit exhaustive enumeration. Steadily increasing computer power and fast virtual screening techniques continue to access full combinatorial libraries by the second approach [11]. Here, we investigate the multi-component Biginelli dihydropyrimidine formation [12,13] as a candidate reaction for virtual screening and hit finding by full library enumeration. We use the self-organizing map (SOM [14]) technique as a ‘pharmacophore dictionary’ that helps prioritize virtual compounds for synthesis and testing [15]. The SOM approach has already demonstrated its predictive ability for combinatorial compound library profiling [16-18], as well as target prediction with drug re-purposing as a prominent application [19-21]. This computational method is ‘unsupervised’ and as such it complements ‘supervised’, model-based prediction systems for *in silico* pharmacology [22-24]. Specifically, we evaluate the applicability of a topological pharmacophore descriptor (CATS [25]) in combination with the SOM-based ‘pharmacophore dictionary’ for target class prediction. By synthesizing and testing a compound from the virtual combinatorial library we were able to confirm its predicted target class.

## 2. Experimental Section

### 2.1. Virtual Compound Library

**Scheme 1:**
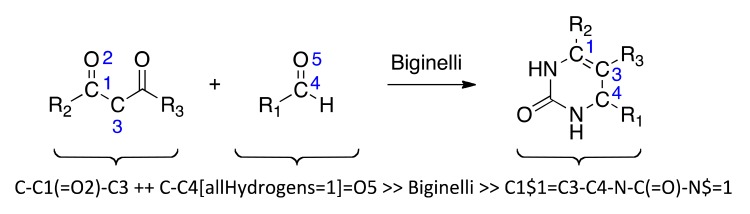
Customized form of the Biginelli reaction and its representation as a *ReactionMQL* string. Blue atom labels indicate the virtual reaction center. Note that the urea is not explicitly listed as one of the educts but appears on the product side.

Biginelli reaction products were enumerated using the *ReactionMQL* toolkit with the reaction represented as ‘reaction string’ (Scheme 1) [26]. Standardization of the virtual educts was done with the software suite MOE (Molecular Operating Environment, v.2010, The Chemical Computing Group, Montreal, QC, Canada) using the ‘wash’ function with default settings. We used the chemical database EXPEREACT (Swiss Federal Institute of Technology, Zurich, Switzerland) as a stock of readily available molecular building blocks for virtual library construction. Building block selection (MW < 300 Da, alog*P* < 2, lack of {Br, I}, single functionality) for the Biginelli reaction yielded 78 aldehydes and 56 diketones. Computational full enumeration resulted in combinatorial library of 4,368 virtual products.

### 2.2. Target Profile Prediction

Topological CATS descriptors [25] were computed for each compound using bin-value scaling by relative frequencies of pharmacophore types [27,28]. This resulted in a 150-dimensional descriptor vector for each molecule, accounting for topological distances between zero and nine bonds, as described elsewhere [28]. The data were projected onto a two-dimensional, toroidal SOM grid. Our SOM implementation *molmap* [29] was used to cluster the COBRA collection of bioactive reference compounds (version 10.3; 11,294 molecules [30]), as described in detail elsewhere (106 training cycles, initial Gaussian neighborhood σ = 7) [20]. The virtual combinatorial compound library was projected onto the trained SOM. Known targets of the COBRA compounds co-located with compound **1** served as a motivation for activity testing.

### 2.3. Synthesis of (N-(4-methoxyphenyl)-6-methyl-2-oxo-4-phenyl-1,2,3,4-tetrahydropyrimidine-5-carboxamide)(**1**)

**Scheme 2:**
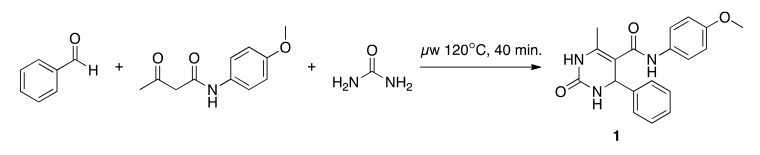
Synthesis of compound **1**.

The Biginelli reaction starts with an acid-catalyzed condensation of the carbamide with the aldehyde. This results in a *N*-acyliminium ion intermediate, which is attacked by the ketone, and through a subsequent cyclization, the dihydropyrimidine product is formed [31,32]. We adapted the synthesis protocol suggested by Stadler and Kappe (Scheme 2) [33]. A 4 M urea (Acros Organics) solution (1 mL, 243.12 mg in 1 mL anhydrous acetic acid) was placed in a microwave vial (size: 2–5 mL, Biotage), and 4 M HCl in dioxane (0.1 mL, 0.4 M) was added as a catalyst. A 10 M benzaldehyde (Acros Organics) solution (0.4 mL, 1.016 mL in 1 mL anhydrous acetic acid) and a 3.3 M *p*-acetoacetaniside (Tokyo Chemical Industry) solution (1.2 mL, 1,370 mg in 2 mL anhydrous acetic acid) were added. The vial was irradiated in the microwave for 40 minutes at 120 °C and stored at 4 °C for 72 hours. The clear colorless solution changed after microwave irradiation to a yellow precipitation. During cooling the precipitation increased.

After vacuum filtration and drying, the raw product was obtained as bright yellow crystals, dissolved in MeOH (4 mL, ultrasonic bath), stored at 4 °C for 18 hours, vacuum filtered and dried in a desiccator over night. The obtained raw product (120.29 mg, yield = 18%) was dissolved in 98% ethyl acetate (3 mL) and MeOH (1 mL). Flash chromatography (flow-rate = 12 mL/min, linear 0–90% ethyl ester/*n*-hexane gradient) was performed on a SNAP cartridge column (KP-SIL 10 g, Biotage). Final product (50.19 mg, yield = 4%) was collected and the solvent was evaporated with nitrogen gas. Purity (area normalization) = 94% (*rt* = 3.14 min), *mp* = 210 °C, *m/z* = 338 (Shimadzu LC-MS2020; HPLC: H_2_O + 0.1% trifluorocetic acid (TFA)/50–95% MeOH + 0.1% TFA, RP18, 250 nm, ESI+); HR-MALDI-MS (Varian IonSpec FT-ICR, 3-HPA): *m/z* = 338.15 (100%, [*M*+H]^+^); ^1^H-NMR (Bruker Avance 400; 516 MHz, DMSO-*d*_6_, proton-proton coupling constants (*J*) are given in Hertz (Hz), ^1^H NMR peak multiplicity is given as s (singlet), d (doublet), t (triplet), m (unresolved multiplet): δ = 9.41 (br. s, 1H), 8.65 (d, *J* = 1.5, 1H), 7.53 (t, *J* = 2.5, 1H), 7.46–7.40 (m, 2H), 7.35–7.20 (m, 5H), 6.84–6.79 (m, 2H), 5.38 (*s*, 1H), 3.69 (s, 3H), 2.02 ppm (s, 3H).

### 2.4. In Vitro Kinase Panel Assay

Compound **1** was analyzed for inhibitory activity against 48 human kinases by Cerep (Cerep, Le Bois l'Evêque, B.P. 30001, 86600 Celle l'Evescault, France; www.cerep.com), *Express Diversity Kinase Profile*, study no. 20507. Two independent assay repetitions (*n* = 2) were performed at a compound concentration of 10 μM.

## 3. Results and Discussion

**Figure 1:**
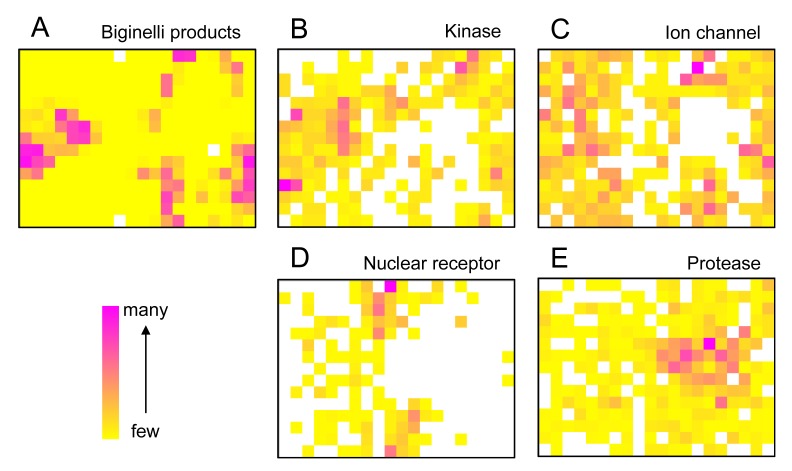
SOM projection of drug-like bioactive compounds (COBRA), with individual target classes highlighted. The SOM grid contains 15 × 20 neurons. In (**A**) the density distribution of Biginelli-type dihydropyrimidines is shown. In (**B**)–(**E**) ligand locations for selected target classes (‘activity islands’) are highlighted.

We started the project by constructing a representation of ‘druglike’ chemical space by training a SOM using the known drugs and lead compounds from the COBRA database. Compounds were encoded by their topological (graph-based, two-dimensional) pharmacophore as computed by the CATS descriptor. Then, we projected a virtual dihydropyrimidine library (4,368 compounds), which we constructed and fully enumerated from available building blocks (78 aldehydes, 56 diketones), onto the SOM. Apparently, the combinatorial products do not fill the whole chemical space defined by the COBRA compounds equally, but seem to be enriched in several patches on the pharmacophore map [Figure 1(A)]. This observation implies that these multi-component reaction products might be suited for distinct target classes rather than binding to all 677 individual drug targets covered by COBRA.

To get a first idea about potential targets for the combinatorial library, we analyzed distributions of target classes on the SOM. It became evident that known kinase inhibitors and ion channel blockers tend to be co-located with the virtual dihydropyrimidines [Figures 1(B,C)], while for example nuclear receptor modulators [Figure 1(D)] and protease inhibitors [Figure 1(D)] might not represent preferred target classes. The observation of ‘activity islands’ in chemical space spanned by topological pharmacophore descriptors is in line with our earlier studies using SOMs for combinatorial library design [34,35].

**Figure 2:**
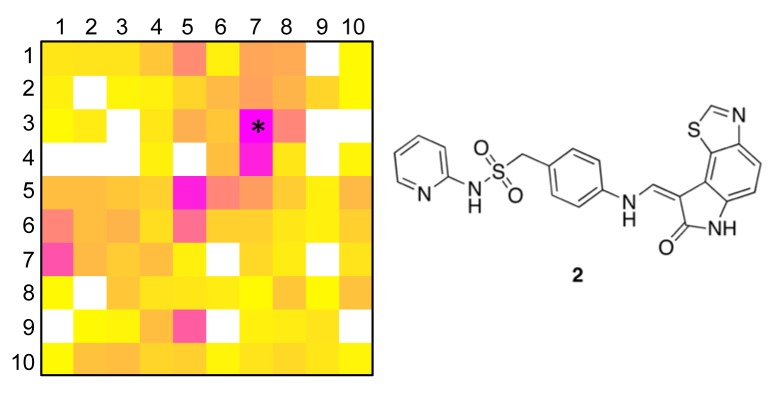
Distribution of known kinase inhibitors on a SOM that was trained with 11,294 drugs and lead compounds (COBRA collection). Coloring indicates prevalence of kinase inhibitors in the 10 × 10 data clusters (*cf.* legend in Figure 1). Compound **1** is located in the most ‘kinase-inhibitor like’ cluster (7/3), marked by an asterisk. Compound **2** is located closest to the cluster centroid represented by the neuron vector.

The distribution of known kinase inhibitors from COBRA is color-coded in the SOM presented in Figure 1. The cluster most densely populated with these reference inhibitors [no. (5/6)] was chosen as target cluster for picking a candidate compound (compound **1**) from the virtual combinatorial Biginelli library for synthesis. Cluster (5/6) contains 29 COBRA compounds with the following target class distribution: 55% kinases, 28% G-protein coupled receptors (GPCR), and 5% proteases. COBRA as a whole contains ligands for 677 individual targets, among which 8% are kinase inhibitors, 33% GPCR ligands, and 15% protease inhibitors. Based on this background distribution we computed a 55/8 ≈ 7-fold over-representation of kinase targets in cluster (5/6), and consequently considerable potential for compounds from this cluster to inhibit kinases. As a check of this prediction, we trained a smaller SOM projection with only 10 × 10 clusters. This means, that all 11,294 COBRA reference compounds were forced into 100 clusters (Figure 2). This compression was performed to reduce the risk of potentially erroneous target predictions due to partially poor sampling in the large SOM. In the small SOM, compound **1** is again located in the dominant ‘kinase-inhibitor like’ field [cluster (7/3), Figure 2], for which kinases are the predicted prevalent targets (4.4-fold over-representation). We conclude that the results from both SOM predictions are consistent.

To obtain a more precise idea of potential kinase targets for compound **1**, we extracted compound **2** as the reference compound from COBRA that is representative for both target clusters [centroid compound for cluster (5/6) in the large SOM, and cluster (7/3) in the small SOM]. Compound **2** is known to potently block cyclin-dependent kinase 2 (CDK2) with an IC_50_ of 10 nM [36].

In order to see whether this hypothesis is correct, we subjected compound **1** to a preliminary activity testing against a panel of 48 activated human kinases (Cerep's *Express Diversity Kinase Profile*). At a concentration of 10 μM compound **1** actually exhibits inhibitory activity against CDK2 (15% of reference inhibitor staurosporine at a concentration of 3.9 nM), MAP/microtubule affinity-regulating kinase 1 (MARK1, 14% of reference inhibitor staurosporine at a concentration of 17 nM), and protein kinase A (PKA, 18% of reference inhibitor staurosporine at a concentration of 6.9 nM), albeit with only moderate activity.

Overall, these results support the usefulness of SOM-based target class prediction based on the CATS descriptor. Inhibitory activity against several human kinases was measured in direct enzyme inhibition assays. Apparently, for the example of CDK2 inhibition, the most confident prediction could be confirmed. It is noteworthy that the chemical structures of compounds **1** and **2** are remakably different, from which it may be rather difficult to deduce a functional relationship (*i.e.*, the same target protein).

**Figure 3:**
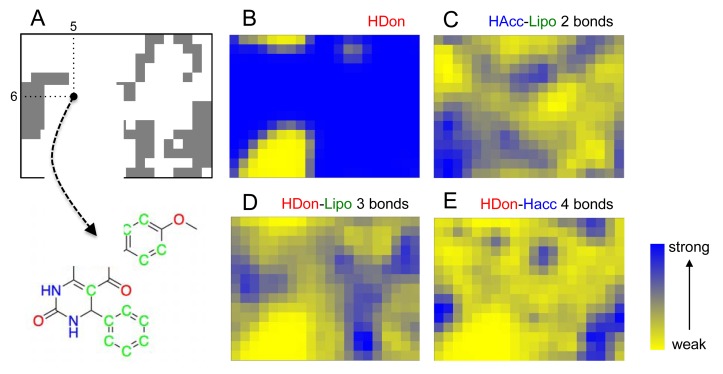
SOM projection of drug-like bioactive compounds (COBRA), with individual CATS features displayed (HDon: *hydrogen-bond donor*, HAcc: *hydrogen-bond acceptor*, Lipo: *lipophilic*). The SOM grid contains 15 × 20 neurons. (**A**) Binary distribution of Biginelli-type dihydropyrimidines. Below, compound **1** from cluster (5/6) is shown with its potential pharmacophoric points highlighted (blue: HDon, red: HAcc, green: Lipo); (**B**)–(**E**) show occurrences of selected CATS descriptors on the SOM.

In addition to cluster visualization and target prediction, the SOM projection offers the possibility to highlight the prevalence of individual pharmacophoric features among the clustered compounds, which can help the medicinal chemist to gain a better understanding of the compound clusters and their relationships, and function as a visual aid (‘pharmacophore dictionary’) for building block selection and molecular design [35]. In Figure 3, the pharmacophore atom-typing for compound **1** is shown, so that CATS descriptors can be matched with the underlying chemical structure. For example, the feature “*hydrogen-bond donor*” (HDon) is present in the majority of the drug-like COBRA compounds and the Biginelli-type dihydropyrimidines (blue regions in Figure 3B). Evidently, this feature alone is insufficient explanation for cluster formation. Additional features are needed, like ‘*hydrogen-bond acceptor spaced two bonds apart from a lipophilic center*’ (HAcc-Lipo 2 bonds, Figure 3C), ‘*hydrogen-bond donor spaced three bonds apart from a lipophilic center*’ (HDon-Lipo 3 bonds, Figure 3D), or ‘*hydrogen-bond donor spaced four bonds apart from a hydrogen-bond acceptor*’ (HDon-HAcc 4 bonds, Figure 3E). Such CATS descriptors can now serve as a guideline for identifying preferred function-determining pharmacophoric features for selected clusters and local areas of the map.

The success of our prediction system may be attributed in part to the ‘fuzzy’ or ambiguous nature of the CATS pharmacophore descriptor. CATS was originally conceived as a molecular representation to facilitate scaffold-hopping in virtual screening and *de novo* design [37]. While such a fuzzy molecular representation appears to be well suited for finding bioisosters and alternative molecular scaffolds, it might not be the appropriate choice for individual target prediction in general [38,39]. This impression is actually supported by comparably poor enrichment of actives in retrospective virtual screening studies with the CATS descriptor [27,39]. Still, due to its coarse-grained nature, it apparently represents a decent choice for first-pass compound library profiling and ligand-based library design as it is sufficiently permissive to accept multiple chemotypes in a target- or target-family focused compound collection [16,30].

The SOM virtual screening approach presented here belongs to the class of ligand-based similarity searching methods [17,24,40]. In contrast to using reference compounds as queries and ranking the combinatorial screening compounds by some pharmacophore similarity index, the SOM offers the potential advantage of performing similarity searching using a ‘common pharmacophore’ model (*i.e.*, the neuron vector) as query. This avoids the necessity for comparing and merging ranked lists of candidate compounds [41,42]. Despite its appeal, the SOM approach used in this study has several disadvantages compared to other ligand-based vistual screening techniques. Most importantly, the choice of the SOM grid layout and topology critically influences compound clustering. Different training runs bear the additional danger of delivering slightly different results due to the stochastic nature of SOM optimization. Several variations and extensions of Kohonen's original SOM algorithm have been published and applied to drug discovery [43]. Recent developments include self-organizing networks with an adapting grid size [44], cascaded SOMs [45], and hybrid neural networks [46,47]. These systems might provide alternative approaches to virtual compound screening, although their practical usefulness and applicability to hit and lead finding still needs to be rigorously assessed. There is no doubt, however, that visualizing compound distributions by two-dimensional graphical displays helps decision making for compound library design and screening candidate selection [48-50].

## 4. Conclusions

The SOM approach to chemical library analysis has been confirmed as a practically applicable tool for compound prioritization and hit identification. The CATS topological pharmacophore descriptor could be corroborated as a molecular representation that allows for valid hypotheses generation about target profiles. The outcome of our study also confirms the concept of topological auto-correlation or properties as a pharmacologically meaningful molecular representation [51,52]. Most likely, less abstract molecular representations than the CATS descriptor, e.g., substructure fingerprints, will be needed to convert the preliminary hit into a validated lead compound by means of computer-assisted design and structure optimization. As compound **1** is relatively small (MW = 337 Da) it qualifies for further optimization [53]. No ADMET warnings were reported when passing it through the FAF-Drugs prediction system [54], and the compound appears to be free of PAINS issues [55,56]. It might also be worthwhile to synthesize and test other Biginelly-type compounds that are co-located with compound **1** in the same SOM cluster.

Judging from the successful integration of the Biginelli-type multi-component reaction to virtual screening, a molecular design cycle seems feasible that is led by a SOM model serving not only as a visual aid in computer-assisted medicinal chemistry, but as a basic ‘pharmacophore dictionary’ for guided lead candidate prototyping. We anticipate combinations of such a tool with fast combinatorial synthesis protocols and compound testing to enable focused compound library design and target profiling with reduced experimental effort.
